# Phospolipase D Insights: Elevated Levels in Mild Cognitive Impairment, Therapeutic Reduction in Alzheimer's Patients

**DOI:** 10.1002/alz.088983

**Published:** 2025-01-09

**Authors:** Cícero Augusto Costa Pereira, Leda Leme Talib, Orestes Vicente Forlenza, Wagner Farid Gattaz

**Affiliations:** ^1^ Laboratory of Neurosciences (LIM27), Departamento e Instituto de Psiquiatria, Hospital das Clínicas, Faculdade de Medicina da Universidade de São Paulo, São Paulo Brazil; ^2^ Laboratório de Neurociências ‐ LIM 27, Sao Paulo Brazil; ^3^ University of São Paulo Medical School, São Paulo Brazil; ^4^ Faculdade de Medicina da Universidade de São Paulo, São Paulo, São Paulo Brazil; ^5^ Laboratory of Neuroscience (LIM‐27), Department and Institute of Psychiatry, Faculty of Medicine, University of São Paulo, São Paulo, São Paulo Brazil; ^6^ University of São Paulo, São Paulo, Brazil, Sao Paulo, São Paulo Brazil

## Abstract

**Background:**

Membrane lipid compromise is also related to AD pathology, a large family of enzymes such as phospholipases that act on membrane integrity, cell signaling, and cellular processes is impaired during the disease phase. Membrane integrity is affected by the B amyloid by the pathological process of AD, moreover, releasing neurotoxic enzymes and vesicles consequently losing important neurotransmitters. Dysregulation of phospholipase D (PLD) can disrupt the plasma membrane and potentially contribute to worsening AD pathology.

**Method:**

The study was conducted at the Institute of Psychiatry, University of Sao Paulo Medical School, Brazil. The plasma samples consisted of Mild Cognitive Impairment (MCI) (n = 20), AD patients (n = 18), and healthy controls (HC) (n = 19). Subjects with other psychiatric or neurological disorders were excluded. PLD activity was measured by a fluorescence method in duplicate by the Amplex® Red Phospholipase D Assay Kit (Thermo Fisher Scientific).

**Result:**

PLD activity showed an increase significantly in the MCL group compared to other groups (p=0.030).

**Conclusion:**

The early stages of dementia have high PLD activity. Our results suggest increased PLD activity significantly in MCL individuals compared to other groups. The highlight its AD with treatment has a decrease in PLD activity compared to other groups.

